# Cough and reflux esophagitis in children: their co-existence and airway cellularity

**DOI:** 10.1186/1471-2431-6-4

**Published:** 2006-02-27

**Authors:** Anne B Chang, Nancy C Cox, Joan Faoagali, Geoffrey J Cleghorn, Christopher Beem, Looi C Ee, Geoffrey D Withers, Mark K Patrick, Peter J Lewindon

**Affiliations:** 1 Department of Respiratory Medicine, Royal Children's Hospital, Herston, Brisbane, Queensland, Australia; 2 Department of Gastroenterology, Royal Children's Hospital, Herston, Brisbane, Queensland, Australia; 3 Department of Anaesthetics, Royal Children's Hospital, Herston, Brisbane, Queensland, Australia; 4 Department of Paediatrics, University of Queensland, Brisbane, Australia; 5 Department of Anatomical Pathology and Cytopathology, Royal Brisbane and Women's Hospital, Herston, Queensland, Australia; 6 Department of Microbiology, Queensland Health Pathology Service, Royal Brisbane and Women's Hospital, Herston, Queensland, Australia

## Abstract

**Background:**

There are no prospective studies that have examined for chronic cough in children without lung disease but with gastroesophageal reflux (GER). In otherwise healthy children undergoing flexible upper gastrointestinal endoscopy (esophago-gastroscopy), the aims of the study were to (1) define the frequency of cough in relation to symptoms of GER, (2) examine if children with cough and reflux esophagitis (RE) have different airway cellularity and microbiology in bronchoalveolar lavage (BAL) when compared to those without.

**Methods:**

Data specific for chronic cough (>4-weeks), symptoms of GER and cough severity were collected. Children aged <16-years (n = 150) were defined as 'coughers' (C+) if a history of cough in association with their GER symptoms was elicited before BAL were obtained during elective esophago-gastroscopy. Presence of esophagitis on esophageal biopsies was considered reflux esophagitis positive (E+).

**Results:**

C+ (n = 69) were just as likely as C- (n = 81) to have esophagitis, odds ratio 0.87 (95%CI 0.46, 1.7). Median neutrophil percentage in BAL was significantly different between groups; highest in C+E- (7, IQR 28) and lowest in C-E+ (5, IQR 6). BAL positive bacterial culture occurred in 20.7% and were more likely present in current coughers (OR 3.37, 95%CI 1.39, 8.08). Airway neutrophilia (median 20%, IQR 34) was significantly higher in those with BAL positive bacterial cultures than those without (5%, 4; p = 0.0001).

**Conclusion:**

In children without lung disease, the common co-existence of cough with symptoms of GER is independent of the occurrence of esophagitis. Airway neutrophilia when present in these children is more likely to be related to airway bacterial infection and not to esophagitis.

## Background

Adult data suggest that gastroesophageal reflux (GER) disease (GERD) causes 21–41% of chronic cough [1,2]. In adults with chronic cough, a small study (n = 8, median % of neutrophils 6.2) has shown a non significant increase of airway neutrophils when compared to controls (n = 10; median % of neutrophils 3.2) [3]. In children with underlying respiratory problems, chronic cough and airway neutrophilia has been reported to occur with GERD [4]. However, there are no prospective studies that have examined the specific relationship between cough and objectively defined GERD in children without an underlying lung disease, and paediatric cough differs significantly from adult cough [5]. Furthermore, cough may exacerbate GER, these common symptoms may simply co-exists and, cause and effect is far from proven [6,7]. Cough is indeed the most common symptom presenting to general practitioners [8,9] and in children, the aetiology of chronic cough is heterogenous [5]. Some studies on children with chronic cough have described airway neutrophilia [10,11] that is possibly related to persistent airways infection [10].

GERD is defined as symptoms or complications of GER [12]. The spectrum of GERD includes reflux esophagitis (RE) and the presence of non-acid reflux, in association with appropriate symptoms [12,13]. Investigations used to confirm and elucidate the diagnosis of GERD includes, histological examination of esophageal biopsies, pHmetry, esophageal manometry and intraluminal impedance monitoring [14]; each modality has its strengths and weaknesses [12,14]. Histological confirmation of RE is a specific way of confirming GERD even though the majority of children with clinical symptoms and complications suggestive of GERD have normal biopsies [12]. pHmetry is said to be more sensitive for acid reflux, but it may not be useful for children [12] with respiratory symptoms and many paediatric gastroenterologists argue that pHmetry alone cannot be used to diagnose GERD. Until intraluminal impedance become more widely available and normal values are known for children, flexible gastrointestinal endoscopy and oesophageal biopsy gives the most precise indication that GER has caused disease (GERD). Flexible upper gastrointestinal endoscopy (esophago-gastroscopy) is the most commonly used investigation for this purpose in some institutions like ours. In order to avoid the inclusion of patients diagnosed with GERD based upon the unproven causality of their symptoms (table 1) by GER, we restricted the study to the evaluation of patients with and without RE.

**Table 1:** Questions on cough and GERD symptoms

	Weighted Kappa
	
**For each symptom, please circle if cough are related**	(95% CI)
A. Heartburn	0.85 (0.60, 1)
always/sometimes/occasionally/never	
B. Vomiting or regurgitation without any cause	0.89 (0.69, 1)
always/sometimes/occasionally/never	
C. Regurgitation that is swallowed (seen or heard)	0.89 (0.61, 1)
always/sometimes/occasionally/never	
D. Difficulty in swallowing	1.0
always/sometimes/occasionally/never	
E. Wakes up at night with pain or abdominal discomfort	0.71 (0.38, 1)
always/sometimes/occasionally/never	
F. A bitter taste in the mouth or pooling of saliva in the mouth	0.79 (0.59,1.0)
always/sometimes/occasionally/never	

In children undergoing esophago-gastroscopy who do not have underlying cardiopulmonary disease (other than mild asthma), the aims of the study were to (1) define the frequency of cough in relation to symptoms of GER, (2) examine if children with cough and RE have different airway cellularity and microbiology in bronchoalveolar lavage (BAL) when compared to those without. We hypothesised that in children without an underlying lung disease, cough is more likely to be present in children with RE than those without RE and, are more likely to have airway neutrophilia.

## Methods

### Subjects

Children aged 0.8–16 years undergoing elective esophago-gastroscopy were invited to participate in the study during the study period (September 2002 till May 2004). All children undergoing esophago-gastroscopy had seen a consultant paediatric gastroenterologist and the procedure performed under general anaesthesia including endotracheal intubation. The primary indication(s) for the esophago-gastroscopy were recorded by the consultant gastroenterologist performing the esophago-gastroscopy. Suspicion of clinical GERD warranting investigation by endoscopy was determined by the consultant paediatric gastroenterologist based upon a history including typical features such as frequent regurgitation, acid brash, nausea, heartburn and/or meal related discomfort. Children were enrolled for the study on the morning of their elective procedure and were opportunistically enrolled i.e. parents of children were approached and recruitment occurred only when the researcher (AC) was able to attend the procedural list. Medical history was obtained from a parent on a standardised proforma for all children and spirometry (Kit, Cosmed, Italy) was performed on those aged ≥6 years. Spirometry values were expressed as percentage predicted, based on Australian data [15]. Parent(s) also scored their child's current cough on a validated cough visual analog scale of 1 (no cough) to 10 (most severe cough) [16]. To determine repeatability of the questions relating cough to GERD (table 1) symptoms, parent(s) of 10 children answered the same questions again within 2–3 weeks. Exclusion criteria were children with; neuro-developmental abnormalities, clinical history of primary aspiration and known underlying cardiorespiratory disease other then mild asthma (no exacerbations in the last 12 months).

Children were categorized as coughers (C+) and non coughers (C-), with reflux esophagitis (E+) and without (E-). GERD was considered present if histology of oesophageal biopsy showed reflux esophagitis (basal cell hyperplasia and mucosal inflammatory neutrophilic infiltrate, with ≤5 eosinophils per high power field) as determined by pathologists blinded to the child's respiratory history [17]. Children were defined as 'coughers' (C+) if the parents or consultant (gastroenterologist or respiratory paediatrician) had elicited a history of chronic cough (>4-weeks) [18,19] with any GERD symptoms (table 1) and scored ≥2 on the cough visual analog scale [16]. Children who had cough on the day of the esophago-gastroscopy were defined as current coughers, which consisted of children with chronic cough and those with a recent (≤4-weeks) history of cough. Written consent was obtained and the study approved by our institution's human ethics committee.

### Bronchoalveolar lavage (BAL)

A non-bronchoscopic standardised and repeatable [20] technique was utilised to obtain BAL fluid, as previously described [21]. Briefly, with the child's head turned to the left, an 8F catheter was passed through the endotracheal tube, beyond the carina. Two specimens were obtained; the first used for microbiology examination and the 2^nd ^collection utilised for cytology. Cell count was performed on the cell suspension, cytocentrifuge slides were prepared and stained (modified Wright's stain) for cell differential profile; additional slides were prepared for lipid laden macrophage index (LLMI) using Oil Red O stain where 100 macrophages counted and scored 0–4. LLMI (range 0–400), as described [22]. All LLMI and cellular examinations were performed by cytologists blinded to the children's medical history.

Quantitative aerobic cultures of bacteria were undertaken on BAL as previously described [22]. Positive bacterial culture was defined as growth of ≥10^4 ^colony forming unit/ml [23,24]. Viral studies were also performed on BAL; direct immunofluorescence antigen (DFA) was used to detect RSV, adenovirus, parainfluenza viruses 1,2,3 and influenza A and B. When DFA was negative, polymerase chain reaction (PCR) tests [25] were undertaken for all the above viruses.

### Statistical analysis

Chi square was used to compare categorical variables between groups and odds ratio described. Data were not normally distributed and non parametric analyses were used; Mann-Whitney for comparisons between 2 groups; Kruskal-Wallis when >2 groups were compared and medians and inter-quartile range (IQR) for descriptive data. Weighted kappa was used to assess agreement. Two tailed p value of <0.05 was considered significant. SPSS ver 11 was utilized for statistical calculation.

## Results

The median age of the 150 children (91 boys, 56 girls) studied was 8.2 years (IQR 7); 163 parents were approached (response rate of 92%). Spirometry was normal in all children who could perform spirometry (n = 81), median FEV_1 _98.6% predicted, FVC 98.5% predicted. Primary indications for esophago-gastroscopy were; abdominal pain (n = 77), recurrent vomiting (n = 35), poor weight gain (n = 20), review of previous lesion (n = 19) and choking (n = 17); some children had more than one indication. Most (n = 136, 90.7%) children were clinically suspected of having GERD and esophagitis was present in 77 (51.3%) children. Only 17 children had tobacco smoke exposure and as numbers were small, comparisons were not made.

Sixty nine (46%) children had chronic cough (C+). In the C+ group, cough has been present for a median length of 52 weeks (IQR 141), median cough score was 4.5 (IQR 3) and there was no difference in cough score between E+ and E- groups, p = 0.88. C+ and C- were equally likely to have RE, odds ratio 0.87 (95%CI 0.46, 1.7). Of the questions relating cough to GERD symptoms, none were associated with the presence of RE (p range from 0.13 to 0.77). The weighted kappa values on the questions relating cough to GERD symptoms (table 1) were between 0.71–1.

**Figure 1:**
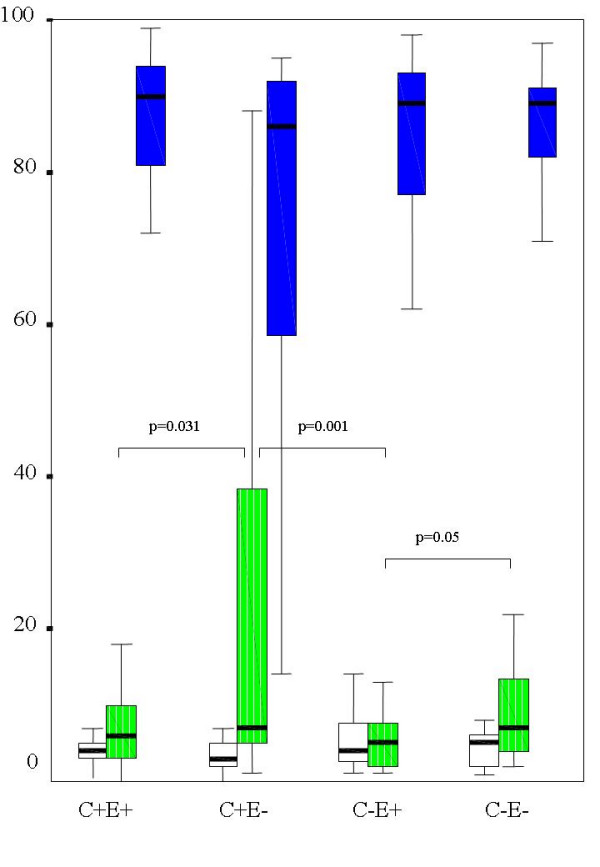
Median and inter-quartile range of BAL cellular profile of all children grouped by cough and reflux esophagitis. The percentage of neutrophils was significantly different between groups; C = cough, E = reflux esophagitis, + = present; - = absent.  Lymphocytes  Neutrophils  Macrophages.

**Figure 2:**
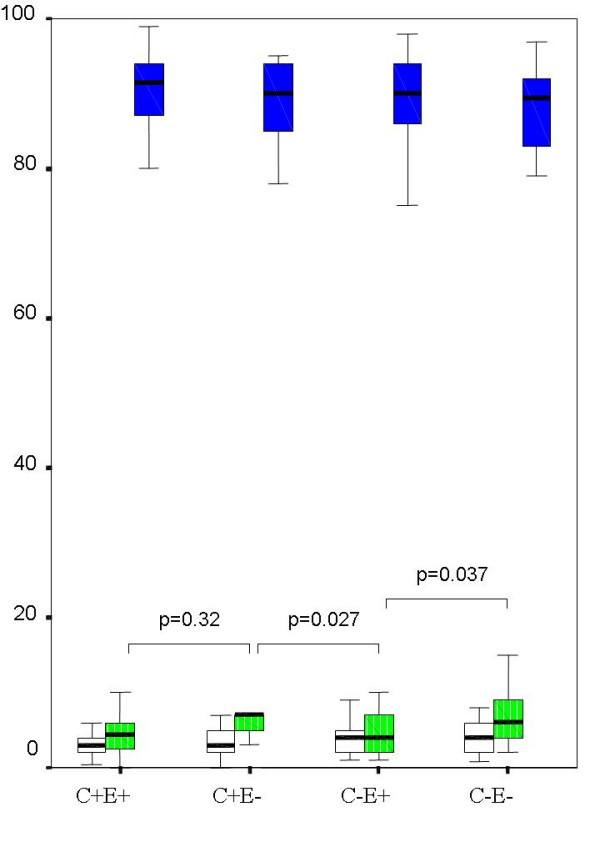
Median and inter-quartile range of BAL cellular profile of children whose BAL did not show positive culture (≥10^4 ^cfu/ml) in the BAL. Children were grouped by presence/absence of cough and RE; C = cough, E = reflux esophagitis, + = present; - = absent. The percentage of neutrophils was significantly different only between selected groups.  Lymphocytes  Neutrophils  Macrophages.

There was no significant difference in cellular profile (percentages of neutrophils, macrophages, lymphocytes and eosinophils in BAL) between C+ (n = 69) and C- (n = 81) groups (p range 0.06 to 0.32). When grouped by presence of cough and GERD, there was a small (table 2) but significant difference in BAL neutrophils % between groups (figure 1, p = 0.008), with the highest values in C+E- group and the lowest in C-E+ group. When children whose BAL showed positive bacterial culture were excluded (n = 31), there was again no difference between C+ (n = 50) and C- (n = 69) in all cellular profile, p range from 0.17 to 0.41. When these children (BAL culture negative) were grouped by presence/absence of cough and GERD, there was no longer any significant difference among groups (p range from 0.06 to 0.76). In 2 group comparisons however, BAL neutrophil % (figure 2) was significantly higher in the C+E- group (n = 21, median 7, IQR 4) compared to C-E+ (n = 38, median 4, IQR 5) groups and C-E- groups (n = 31, median 6, IQR 5.2). BAL neutrophil % was also significantly higher in C-E- when compared to C-E+.

**Table 2:** Cellular profile of children grouped by presence (+) and absence (-) of cough (C) and Reflux Esophagitis (E)

	**C+E+ **N = 33	**C+E- **N = 36	**C-E+ **N = 44	**C-E- **N = 37	p*
Lymphocyte %					
Median, IQR	4, 1.3	3, 4	4, 5	5, 4	0.486
Neutrophil %					
Median, IQR	6, 7.6	7, 28	5, 6	6.5, 9.3	**0.007**
Macrophage %					
Median, IQR	90, 13.5	86, 34	89, 16	88.7, 10.8	0.629
Eosinophil %					
Median, IQR	0, 0	0, 0	0, 0	0, 0	0.514
Total cell count					
Median, IQR	114, 160	138, 184.5	93, 190.5	104, 114	0.098
LLM index					
Median, IQR	33, 53	37, 31.5	47.5, 37	38, 25	0.188

*p value by Kruskal Wallis

LLM = lipid laden macrophage, IQR = inter-quartile range

Positive bacterial cultures was present in BALs of 31 children (20.7%); *S. pneumoniae *in 19 children, *H. influenzae *in 10, *M. catarrhalis *in 7 and *S. aureus *in 3 children (some had >1 bacteria type). Of the 31 children with positive bacterial culture in their BAL, 23 had current cough, and 19 were in C+ group. The cellular profile of these 31 children (median %neutrophils was 20, IQR 34; %lymphocytes 6, 8.5; %macrophages 65.5, 34) was significantly different to those without positive BAL culture (%neutrophils 5, 4; %lymphocytes 3, 4; %macrophages 90, 9); p of 0.00001 for all cell types. Positive BAL cultures was more likely to be present in children with current cough than in children without current cough but there was no significant difference between C+ and C- groups, and between E+ and E- groups (table 3). Viral studies (DFA and PCR) were negative in all the BAL samples.

**Table 3:** Comparisons of groups with positive bacterial culture in BAL

	BAL culture			
Group category	Negative (<10^4 ^cfu/ml)	Positive (≥10^4 ^cfu/ml)	p value	OR, 95% CI
No current cough	64	8		
Current cough	55	23	0.005	3.35, 1.39–8.08
C-	69	12		
C+	50	19	0.055	2.19, 0.97–4.91
E-	52	17		
E+	67	14	0.270	0.64, 0.29–1.42

## Discussion

In children without an underlying lung problem (other than mild asthma), cough was commonly present in association with gastro-intestinal symptoms suggestive of GERD. However cough was just as likely to be present in children with and without RE and, none of the common symptoms of GERD with cough was associated with the presence of RE. Airway neutrophilia was highest in children with cough and without RE (C+E-) and, lowest in children without cough and with RE (C-E+). Positive bacterial culture with recognised respiratory pathogens was relatively common at 20.7%, more likely to occur in current coughers but, equally present in children with and without RE.

Given that it is controversial whether GERD is a significant cause of respiratory illness (as opposed to co-existing) [6], it is not surprising that relating the common symptom of cough to GERD is also controversial. In our cohort of children presenting to gastroenterologists for evaluation of GER as opposed to children selected from respiratory clinics, children with RE were as likely as children without RE to have a history of cough. Studies based on children from respiratory clinics have shown a high frequency of GERD [26-29]. All these studies described that GER (diagnosed by ambulatory pHmetry [26,29] and other investigations including esophago-gastroscopy [27,28]) was a common cause and/or contributor of the children's respiratory symptoms that included chronic cough, asthma, and upper airway symptoms (stridor, laryngitis). Cohort studies on highly selected patients almost invariably show a positive association between GER and respiratory symptoms. Indeed there is only one published paediatric study (Pubmed search 5/7/05) that has detailed no association between respiratory symptoms and GER [30]. In contrast, systematic reviews especially in evidence based treatment effects on GERD and respiratory symptoms has shown that the evidence for the linkage is significantly weaker than published cohort studies [31]. Indeed, using Cochrane methodology we found a low effect of GERD treatment on cough [32] which is in contrast to non-controlled trials where the cough improvement rates by non-surgical intervention was as high as 86–100% [33,34].

The coexistence of symptoms do not imply causation [6,7]. Like asthma and GER, cough and GER are also common and thus "would be expected to coexist, purely on the basis of chance" [7]. None of the studies that showed an association between GERD and respiratory symptoms in children with an underlying respiratory problem had adequate controls and cough itself could lead to reflux events [35,36]. Using multichannel intra-luminal impedance monitoring in adults, Sifrim and colleagues recently showed that cough leading to reflux occurred in half of the episodes (as opposed to reflux leading to cough) [36].

Chronic cough in children is heterogenous [5,19]. Some paediatric cohort studies have shown that GERD is rarely (3–8%) [37,38] the cause of cough. We found that bacteria bronchitis (based on positive BAL culture) associated with neutrophilia was common and significantly more likely to be present in children with cough [39]. Similarly, Fitch et al described that airway neutrophilia was common in children with non-specific chronic cough and speculated that it was likely related to persistent airways infection [10]. We defined positive bacteria culture on a threshold of ≥10^4 ^cfu/ml based on previous studies [17]. However, the diagnostic threshold for quantitative culture on BAL for bronchitis in children (as opposed to pneumonia) is unknown and caution is needed in the interpretation of BAL microbiology [40].

This study was neither a controlled trial nor one designed to define the aetiology of cough. In the context of the methodological problems in studying cough in children that includes the relative unreliable subjective reporting of cough (when compared to objectively measured cough [16,41] and large placebo and time period (natural resolution with time) effects [32,42], attributing cough to an aetiology in a non controlled study is fraught with errors. We thus chose not to look at effect of any 'treatment' on cough. We cannot be definitive, but our findings of high occurrence of significant bacteria culture also suggest that chronic cough in children are unlikely caused by RE alone.

Postulated mechanisms between cough and GERD include aspiration and sensory nerve stimulation [43]. We have previously shown that E+ (called G+ in previous paper) group had the lowest airway neutrophils [21] as also described in a small (n = 11) study in adults with GERD and cough [44]. Our findings showing airway neutrophilia highest in the C+E- group suggests that although cough was identified in association with GERD symptoms, the aetiologies of the cough, in most children is unlikely to be related to RE alone. As we had described that airway microbiology do not reflect gastric aspirates in these children, aspiration of gastric contents is very unlikely cause of the airway neutrophilia [21]. In contrast, Sacco et al described increased airway neutrophilia and LLMI in children with GERD and respiratory symptoms [4]. They however diagnosed GERD based on pHmetry, enrolled children from a different setting to ours as they selected children from respiratory clinics and, did not examine microbiology of the airways [4]. Bacterial airway infections causes airway neutrophilia and this is likely the main reason why neutrophilia was highest in the C+ group. Other causes or contributors to airway neutrophilia are however possible and this cannot be addressed in our study. Adult studies on chronic cough have found a significant number of patients with airway eosinophilia [3] but our study like other paediatric studies [10,45,46] demonstrated that airway eosinophilia is a very infrequent finding in children with chronic cough without an underlying pulmonary disease.

Our data cannot be extrapolated to other definitions of GERD and it is possible that other definitions of GERD may yield different findings. Thus while the discussions have so far emphasised that the symptom of cough in children with GER symptoms is either poorly related to RE, it is also possible that esophago-gastroscopy is less useful investigatory tool for paediatric cough. However, there is no single perfect method for the objective definition of all GERD types and arguably, esophagitis is the gold standard definition as stated by the AGA guidelines "In the absence of esophagitis, there is no gold standard for the definition of GERD..." [13]. We used esophageal biopsy as the definition for practical reasons and it is also a common method of diagnosing GERD in Australian children. pHmetry has been reported to be more sensitive but there is considerable disagreement on what constitutes an abnormal pHmetry for cough associated GERD and, acid reflux occurs in normal people [12,13,47]. Assertions that cough is related to acid GERD can occur with unique pHmetry indices (eg reflux index of 0% required to be cough free) and that cough can take a prolonged time (a year) to settle post GERD intervention [48] are difficult to prove or disprove in the context of the difficulties with using cough as the primary outcome measure in studies and the feasibility of the required studies. The controversy of extra-esophageal manifestations of GERD [49] such as laryngopharyngeal reflux also is beyond the scope of this article. Review articles and guidelines [12,49] are available; in the North American Society for Pediatric Gastroenterology and Nutrition guidelines for paediatric GER article, the section on upper airway symptoms included a discussion on cough and GER. The conclusion reached in the section was "...there is insufficient evidence and experience in children for a uniform approach to diagnosis and treatment" [12].

In addition to the limitation of our GERD definition, our study is also limited by the small sample size on the repeatability of the questions relating cough to GERD symptoms. The good repeatability found was however consistent with existing data; questions on cough combined with another symptom has good repeatability [42,50,51]. Furthermore, we did not use a standardised research based questionnaire for infant or child detection of GER such as those devised by Orenstein [52] because of its non feasibility in the context of the study. Lastly another potential limitation of the study is the use of blind BAL. However this method is widely used [53] for practical reasons and the cellularity values in our C-E- group were similar to published data on bronchoscopically obtained BAL [40].

## Conclusion

We conclude that, in children without lung disease the common co-existence of cough with symptoms of GERD is independent of the occurrence of RE. Airway neutrophilia when present in these children were more likely to be related to airway bacterial infection and not to RE. Further objective and in-depth studies are required to evaluate the common subjective occurrence of cough in children with GER symptoms.

## Abbreviations

BAL: bronchoalveolar lavage

C+: coughers = if a history of cough in association with their GER symptoms was present

C-: non coughers

DFA: direct immunofluorescence antigen

E: esophagitis

FEV_1_: forced expiratory volume in one second

FVC: Forced vital capacity

GER: gastroesophageal reflux

GERD: gastroesophageal reflux disease

IQ:R inter-quartile range

LLMI: lipid laden macrophage index

PCR: polymerase chain reaction RE: reflux esophagitis

## Competing interests

The author(s) declare that they have no competing interests.

## Authors' contributions

AC conceived the idea, designed the study, performed the data analysis and drafted the manuscript. JF and NC designed the microbiology and cytological components respectively and both helped draft the manuscript. GC, LE, GW, CB, MP and PL participated in the study design, data acquisition and writing of manuscript. All authors read and approved the manuscript.

## Pre-publication history

The pre-publication history for this paper can be accessed here:


